# Combination of COX-2 expression and *PIK3CA* mutation as prognostic and predictive markers for celecoxib treatment in breast cancer

**DOI:** 10.18632/oncotarget.13200

**Published:** 2016-11-08

**Authors:** Sandrine Tury, Véronique Becette, Franck Assayag, Sophie Vacher, Camille Benoist, Maud Kamal, Elisabetta Marangoni, Ivan Bièche, Florence Lerebours, Céline Callens

**Affiliations:** ^1^ Pharmacogenomic Unit, Genetics Laboratory, Institut Curie, Paris, France; ^2^ Department of Pathology, Institut Curie, Hôpital René Huguenin, Saint-Cloud, France; ^3^ Laboratory of Preclinical Investigations, Translational Research Department, Institut Curie, Paris, France; ^4^ Department of Medical Oncology, Institut Curie, Paris and Saint-Cloud, France

**Keywords:** breast cancer, PIK3CA, celecoxib, prognosis, predictive biomarker

## Abstract

COX-2 expression level and prognostic value are still a matter of debate in breast cancer (BC). We addressed these points in the context of *PIK3CA* mutational status. Based on an interesting study of aspirin efficacy in colorectal cancer, we hypothesized that celecoxib antitumoral activity may be restricted to *PIK3CA* mutated BC.

*COX-2* mRNA expression was analyzed in 446 BC samples and in 61 BC patient-derived xenografts (PDX) using quantitative RT-PCR. The prognostic impact of COX-2 expression level was assessed independently and according to *PIK3CA* mutational status in our cohort and in a validation set of 817 BC. The antitumoral activity of celecoxib was tested in two triple-negative (TN) PDX with a *PIK3CA* wild-type (wt) or mutated genotype.

*COX-2* mRNA was overexpressed in 2% of BC and significantly associated with TN subtype. Metastasis-free survival (MFS) was significantly better in patients with high *COX-2* expression level, the prognosis of whom was similar to patients with *PIK3CA* mutations. TCGA validation cohort confirmed that patients with low *COX-2* expression *PIK3CA* wt tumors had the worse disease-free survival (DFS) compared to all other subgroups. Celecoxib had a significant antitumoral effect in *PIK3CA* mutated PDX only. Celecoxib antitumoral activity involved S6 ribosomal protein and AKT phosphorylation.

Low expression of *COX-2* has a significant negative impact on the MFS/DFS of BC patients. Antitumoral effect of celecoxib is restricted to *PIK3CA* mutated PDX. These results suggest that *PIK3CA* mutation may be a new predictive biomarker for celecoxib efficacy.

## INTRODUCTION

The cyclooxygenase-2 (COX-2) also known as the prostaglandin-endoperoxide synthase-2 (PTGS-2) is an inducible enzyme involved in inflammatory and oncogenic processes. It is responsible for the synthesis of prostaglandins from arachidonic acid [1] and is reported to induce the expression of aromatase in breast tissue [2, 3]. COX-2 expression level in breast carcinomas and normal breast tissue is not well established and reports are controversial. COX-2 expression levels in ductal carcinoma *in situ* (DCIS) and invasive carcinoma were reported to be similar in a meta-analysis of COX-2 expression levels in breast cancers (BC). No clear conclusion on COX-2 expression levels in normal breast epithelium was however reported in the latter study [4]. A recent study using immunohistochemistry (IHC) assessed COX-2 expression on BC and adjacent normal tissues from 96 premenopausal women. COX-2 expression in the normal breast epithelium fluctuated (more than 40-fold) among women and was correlated with COX-2 expression levels in DCIS and invasive cancer, independently of known prognostic features. The authors suggested that factors regulating physiological COX-2 expression may be the primary drivers of COX-2 expression in BC. Thus, baseline COX-2 expression level may be an indicator of BC risk, and predict chemo preventive and therapeutic efficacy of COX-2 inhibitors in young women [5]. The prognostic value of COX-2 is still debated. Several studies suggested that COX-2 is implicated in BC progression, where COX-2 overexpression was shown to be associated with poorer outcome. On the other hand, this negative prognostic impact may be counterbalanced by hormonal treatment [6–10].

Giving the putative prognostic role of COX-2, the potential therapeutic benefit of COX-2 inhibitors has been investigated. Celecoxib, a non-steroidal anti-inflammatory drug (NSAID), is a specific COX-2 inhibitor. Celecoxib acts mainly by decreasing the formation of downstream target proteins prostaglandin, prostacyclin or thromboxane involved in cell proliferation and angiogenesis. Celecoxib has thus been examined for its antitumoral properties [11, 12].

*In vitro* and *in vivo* studies have shown an antitumoral effect of celecoxib in BC. Celecoxib significantly decreased tumor volume by 32% in rats with chemically induced mammary tumor [13]. Celecoxib was also reported to significantly decrease tumor incidence rate and delayed tumor emergence in similar animal models [14].

Although preclinical data were optimistic, the clinical trials results testing the efficacy of celecoxib in BC patients were disappointing. The combination of celecoxib and aromatase inhibitors was tested in clinical trials since celecoxib may enhance aromatase inhibitors' efficacy. In DCIS, two studies have led to conflicting results [15, 16]. In a phase II trial for advanced BC women with progressive disease under tamoxifen, celecoxib in association with exemestane did not improve clinical outcome as compared to exemestane alone [17]. Another multicentric randomized phase II study of neoadjuvant epirubicin/cyclophosphamide followed by docetaxel (EC-D) with or without celecoxib showed that celecoxib is not likely to improve complete pathological response rates in addition to EC-D in patients with HER2-negative tumor [18]. A trial on 90 DCIS postmenopausal patients with ER-positive carcinoma showed that two weeks presurgical treatment with celecoxib alone or in combination with exemestane had no effect on proliferation or apoptose [15]. More recently, a monocentric phase II neoadjuvant trial in postmenopausal women with ER-positive DCIS (n=95) showed that concomitant administration of celecoxib and exemestane during 12 weeks induced a significant reduction in tumor cell proliferation and COX-2 expression. These results suggest that COX-2 high expression levels may be a predictive marker for early relapse in patients with DCIS and may help in the clinical decision for treatment of DCIS [16]. Despite the different encouraging results, it remains to be established whether BC patients might actually benefit from celecoxib treatment.

In 2012 Liao *et al*. showed that aspirin, a non-selective COX inhibitor, increased overall survival in patients with colorectal cancer harboring an activating mutation in the *PIK3CA* gene. These results substantiate an interaction between the cyclooxygenase activity and the PI3K/AKT pathway [19]. Other studies confirmed the benefit of aspirin treatment on overall survival in *PIK3CA* mutated colorectal cancer [20]. As *PIK3CA* mutations are reported in 10-40% of BCs [21] we hypothesized that mutated-*PIK3CA* breast tumor expressing COX-2 could benefit from treatment with a COX-2 inhibitor such as celecoxib.

In the present study we first evaluated COX-2 expression levels and prognostic value according to the *PIK3CA* mutational status in a large retrospective cohort of BC patients. We then investigated the antitumoral effect of celecoxib depending on *PIK3CA* mutation in triple-negative patients-derived xenograft models (PDX). Finally, we assessed potential predictive biomarkers and secondary resistance mechanisms associated with celecoxib antitumoral properties.

## RESULTS

### *COX-2* overexpression is rare and associated with TNBC

We first quantified the expression level of *COX-2* transcript by quantitative RT-PCR (qRT-PCR) in a cohort of 446 BC samples composed of 68 HR^-^ERBB2^-^, 42 HR^-^ERBB2^+^, 285 HR^+^ERBB2^-^ and 51 HR^+^ERBB2^+^ cases.

**Figure 1:**
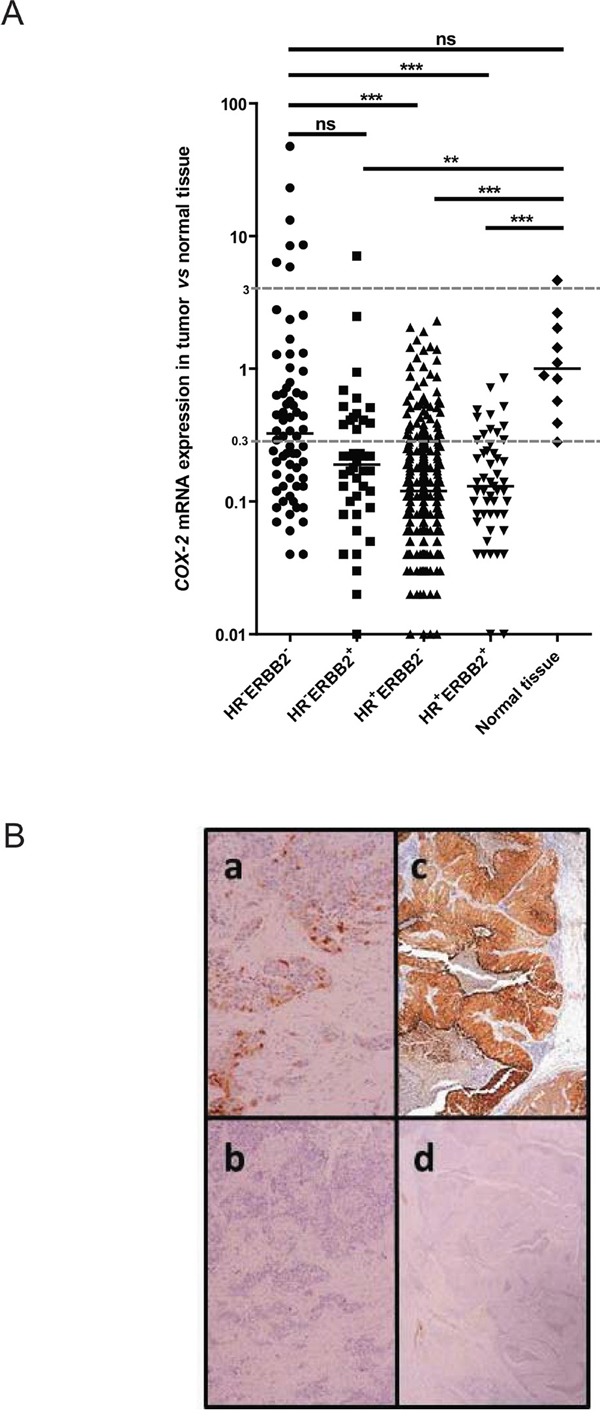
*COX-2* mRNA and protein expression in patient breast tumors **A.** *COX-2* mRNA expression levels in 446 breast tumor samples (68 HR-ERBB2-, 42 HR-ERBB2+, 285 HR+ERBB2- and 51 HR+ERBB2+) and in 10 normal breast tissues using qRT-PCR. HR for hormone receptors. *COX-2* mRNA expression in breast tumor samples is expressed compared to *COX-2* mRNA expression in normal tissue. **B.** COX-2 protein expression in a mRNA *COX-2* expressing tumor, weak (a) and strong (c). b and d: the same tumors stained with the isotypic control antibody.

*COX-2* transcript was underexpressed (relative expression <0.3 compared to normal tissue as detailed in material and method section) in 74% (332/446) and overexpressed (relative expression >3 compared to normal tissue) in 2% (8/446) of cases. By comparison with normal tissue *COX-2* mRNA relative expression was significantly higher in triple-negative subtype than in HR+ERBB2- and in HR+ERBB2+ subtypes (Figure 1A). This result was identical when evaluating *COX-2* mRNA expression without normal tissue comparison (Supplementary Figure S1A). Considering the relative expression cut-off of 3, *COX-2* overexpression is strongly associated with the triple-negative subtype (10%, 7/68 tumors, p<0.0001)

*COX-2* transcript was also quantified by qRT-PCR in 61 tumors collected on PDX (15 HR^+^, 6 ERBB2^+^ and 40 triple negative tumors). In PDX, the strongest expression levels of *COX-2* were found in triple-negative (median 36 [0–1673]) compared to luminal (median 0 [0–202]) (p=0.0006) and ERBB2 positive subtypes (median 6 [0–601]) (p=ns) (Supplementary Figure S1B).

COX-2 IHC staining in 26 primary tumors representative of our cohort revealed no labeling when the expression of *COX-2* transcript was inferior to 2 (relative expression compared to normal tissue) and the staining becomes more intense as the level of expression of transcript increases (Table 1 and examples are shown in Figure 1B). The same observation was made in 14 PDX where COX-2 IHC staining was more intense when *COX-2* transcript was more expressed (Table 2). These data show a good correlation between the *COX-2* mRNA and the COX-2 protein expression levels except for two cases (2/26, 8%) of primary tumor (3395 and 5015). Technical difficulties prevented us from accurately determine the percent of positive cells and intensity of the staining in particular histological types of breast carcinomas like ductal carcinoma in situ (6189), neuroendocrine carcinoma (6602) and metaplastic carcinoma (HBCx-60).

**Table 1:** Correlation between *COX-2* mRNA and COX-2 protein expression level on 26 primary breast tumors

samples ID	*COX-2* mRNA expression	COX-2 protein expression	positive cells (%)	staining intensity
4410	<0.05	negative	0	0
4207	<0.05	negative	0	0
5396	<0.05	negative	60	1
6645	0.03	negative	0	0
5461	0.04	negative	0	0
4393	0.20	negative	0	0
5470	0.20	negative	0	0
6605	0.79	negative	2	2
3395	1	positive (moderate)	25	3
6189	1	negative	80	1
6189^a^	1			
6601	1	negative	80	1
6891	1	negative	0	0
5708	1.47	negative	0	0
2421	1.67	negative	0	0
2690	1.91	negative	0	0
6602^b^	2.05			
5295	2.53	positive (moderate)	50	2 to 3
5295	2.53	positive (moderate)	20	2 to 3
5015	5.85	negative	0	0
6874	6.33	positive (moderate)	40	2 to 3
6889	7.07	positive (high)	70	2 to 3
6889	7.07	positive (moderate)	20	2 to 3
5392	8.46	positive (moderate)	<<1	3
2346	8.58	positive (high)	100	2 to 3
6876	47.31	positive (high)	70	2

a Corresponding to ductal carcinoma in situ (DCIS) case.

b Corresponding to neuroendocrine carcinoma case.

**Table 2:** Correlation between *COX-2* mRNA and COX-2 protein expression level on 14 PDX samples

samples ID	*COX-2* mRNA expression	COX-2 protein expression	positive cells (%)	staining intensity
HBCx-10	0	negative	0	0
HBCx-51	0	negative	0	0
HBCx-22	1	negative	0	0
HBCx-28	7	negative	0	0
HBCx-43	26	negative	0	0
HBCx-16	38	negative	0	0
HBCx-49	38	positive (weak)	1	3
HBCx-30	39	positive (weak)	<1	2
**HBCx-4B^b^**	**218**	**positive (moderate)**	**15**	**2 to 3**
HBCx-23	264	positive (moderate)	5	3
HBCx-8	339	positive (high)	50	2 to 3
HBCx-60**^a^**	504			
**HBCx-52^b^**	**579**	**positive (high)**	**60**	**2 to 3**
HBCx-15	658	positive (moderate)	15	2 to 3

a Corresponding to metaplastic carcinoma case.

b Corresponding to PDX selected for *in vivo* experiments.

### Relation between *COX-2* mRNA level and clinico-pathological parameters

The characteristics of the 446 breast tumors according to the individual *COX-2* mRNA level are shown in Table 3. Age of patients, SBR histological grade, lymph node status, tumor size and *Ki67* mRNA expression were not statistically different in patients with different *COX-2* expression levels. Hormone receptor status was the only parameter associated with *COX-2* mRNA level (ERα: p<10^-4^; PR: p=0.0016) (Table 3).

**Table 3:** Relationship between *COX-2* transcript expression level and classical clinical and biological parameters in a series of 446 breast cancers

	Total population (%)	Number of patients (%)		p value^a^
		*COX-2* mRNA expression <0.3 relative to normals	*COX-2* mRNA expression >0.3 relative to normals	
**Total**	446 (100)	332 (74.4)	114 (25.6)	
**Age**				
**≤50**	94 (21.1)	67 (71.3)	27 (28.7)	0.59
**>50**	352 (78.9)	261 (74.1)	91 (25.9)	
**SBR histological grade^b^^,^^c^**				
**I**	57 (13)	43 (75.4)	14 (24.6)	0.36
**II**	223 (51)	172 (77.1)	51 (22.9)	
**III**	157 (35.9)	111 (71)	46 (29)	
**Lymph node status^d^**				
**0**	118 (26.5)	89 (75.4)	29 (24.6)	0.69
**1-3**	232 (52.1)	169 (72.8)	63 (27.2)	
**>3**	92	71 (77.2)	21 (22.8)	
**Macroscopic tumor size^e^**				
**≤25**	220 (50.2)	166 (75.4)	54 (24.6)	0.62
**>25**	218 (49.8)	160 (73.4)	58 (26.6)	
**ERα**				
**Negative**	115 (25.8)	64 (55.6)	51 (44.4)	**<10^-4^**
**Positive**	331 (74.2)	264 (79.7)	67 (20.3)	
**PR**				
**Negative**	190 (42.6)	125 (65.8)	65 (34.2)	**0.0016**
**Positive**	256 (57.4)	203 (79.3)	53 (20.7)	
**ERBB2**				
**Negative**	353 (79.1)	263 (74.5)	90 (25.5)	0.95
**Positive**	93 (20.9)	69 (74.2)	24 (25.8)	
**Subgroups**				
**HR-ERBB2-**	68 (15.2)	32 (47)	36 (53)	**<10^-4^**
**HR-ERBB2+**	42 (9.4)	28 (66.7)	14 (33.3)	
**HR+ERBB2-**	285 (63.9)	231 (81.4)	54 (18.6)	
**HR+ERBB2+**	51 (11.4)	41 (80.4)	10 (19.6)	
***PIK3CA* status**				
**Wild type**	298 (66.8)	208 (69.8)	90 (30.2)	**<10^-4^**
**Mutated**	148 (33.2)	120 (81)	28 (19)	
***Ki67* mRNA expression**				
**Median**	12.4 (0.80-117)	12 (0.80-117)	13.2 (1.05-94.5)	0.42^f^
***EGFR* mRNA expression**				
**Median**	0.22 (0-106)	0.17 (0.02-7.56)	0.47 (0-106)	**<10^4^^f^**
**Metastasis**				
**No**	282 (63.2)	198 (70)	84 (30)	**<10^-4^**
**Yes**	164 (36.8)	134 (82)	30 (18)	

a Chi^2^ test

b Scarff bloom Richardson classification.

c Information available for 437 patients.

d Information available for 442 patients.

e Information available for 438 patients.

f Kruskal Wallis's H test.

We then tested the relation between *COX-2* mRNA expression levels and both *EGFR* mRNA level and *PIK3CA* mutation status, previously determined in these tumor samples [33, 34]. *PIK3CA* mutations were detected in 33% of patients (148/446). *COX-2* mRNA level was tightly linked to *EGFR* mRNA levels (p<10^-4^) and to *PIK3CA* mutations (p<10^-4^) (Table 3).

### Prognostic impact of *COX-2* mRNA expression level

**Figure 2:**
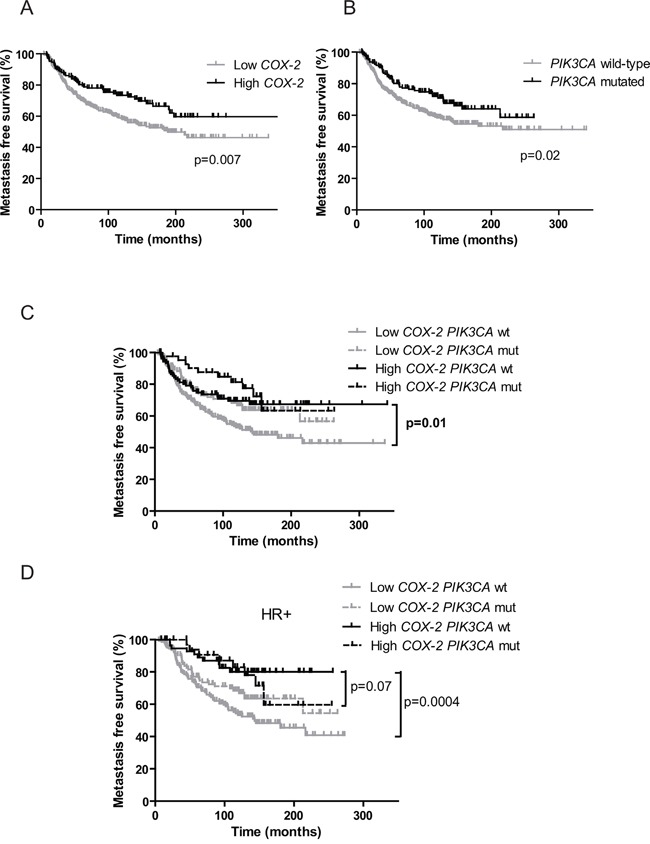
Prognostic value of *COX-2* mRNA expression and *PIK3CA* mutations on patients' metastasis-free survival **A.** Kaplan-Meier estimates of metastasis-free survival according to *COX-2* mRNA expression. **B.** Kaplan-Meier estimates of metastasis-free survival according to *PIK3CA* mutations. **C.** Kaplan-Meier estimates of metastasis-free survival according to *COX-2* mRNA expression and *PIK3CA* mutations in the global cohort. wt for wild-type, mut for mutated. **D.** Kaplan-Meier estimates of metastasis-free survival according to *COX-2* mRNA expression and *PIK3CA* mutations in HR+ patients.

SBR grade (p=1.5.10^-4^), lymph node status (p=1.9.10^-3^), tumor size (p=1.4.10^-5^), ER (p=8.4.10^-6^) and PR (p=8.6.10^-6^) status as well as *PIK3CA* mutations (p=0.02) all had prognostic value as measured by the 5-years MFS. A trend towards a worse MFS among patients with low *COX-2* expression (using optimal cut-off determined as described in material and methods section) was observed (p=0.05) (Table 4). This trend became statistically significant when evaluating the prognostic impact of low *COX-2* expression for the complete follow-up delay of this cohort (p=0.007) (Figure 2A). Multivariate analysis (Cox proportional hazards model) was also used to assess the influence of *COX-2* mRNA level on MFS, together with histological grade, lymph-node status, tumor size, estrogen and progesterone receptor status and *PIK3CA* mutations. Lymph node status >3 (p=0.02), SBR grade III (p=0.04), tumor size >25mm (p=0.02) and low *COX-2* mRNA expression (p=0.01) were statistically associated with poor prognosis (Supplementary Table S1).

**Table 4:** Characteristics of the 446 primary breast tumors and relation to metastasis-free survival

	Number of patients	5 years MFS	p value^a^
**Total**	446	72.6%	
**Age**			
**≤50**	94	70.6%	0.33
**>50**	352	74.6%	
**SBR histological grade^b^^,^^c^**			
**I**	57	92.4%	**1.5.10^-4^^f^**
**II**	223	76%	
**III**	157	64.2%	
**Lymph node status^d^**			
**0**	118	79.4%	**1.9.10^-3^^f^**
**1-3**	232	76.5%	
**>3**	92	59.2%	
**Macroscopic tumor size^e^**			
**≤25**	220	82.9%	**1.4.10^-5^**
**>25**	218	64.1%	
**ERα**			
**Negative**	115	60.2%	**8.4.10^-6^**
**Positive**	331	78.3%	
**PR**			
**Negative**	190	63.5%	**8.6.10^-6^**
**Positive**	256	81.3%	
**ERBB2**			
**Negative**	353	75.1%	0.11
**Positive**	93	68.5%	
**Subgroups**			
**HR-ERBB2-**	68	61.4%	**1.5.10^-5^^f^**
**HR-ERBB2+**	42	53.8%	
**HR+ERBB2-**	285	78.3%	
**HR+ERBB2+**	51	81%	
***PIK3CA* status**			
**wild type**	298	70.4%	**0.02**
**mutated**	148	80.2%	
***COX-2* expression**			
**≤0.22**	294	70.6%	**0.05**
**>0.22**	152	80.1%	

a Log-rank test.

b Scarff bloom Richardson classification.

c Information available for 437 patients.

d Information available for 442 patients.

e Information available for 438 patients.

f Global comparison of all subgroups of a category.

### COX-2 expression presents a prognostic value in *PIK3CA* wild-type patients

**Figure 3:**
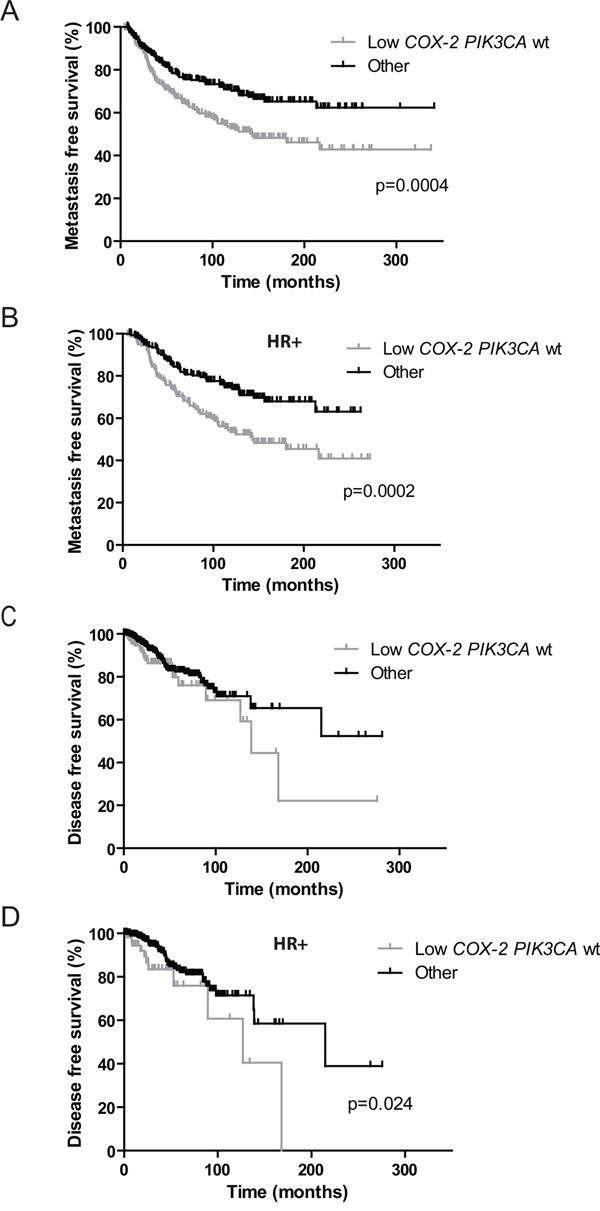
Prognostic value of low *COX-2* mRNA expression and wild-type *PIK3CA* status *versus* other subgroups on patients' metastasis and disease-free survival **A.** Kaplan-Meier estimates of metastasis-free survival according to *COX-2* mRNA expression and *PIK3CA* mutations in the global cohort. **B.** Kaplan-Meier estimates of metastasis-free survival according to *COX-2* mRNA expression and *PIK3CA* mutations in HR+ patients. **C.** Kaplan-Meier estimates of disease-free survival according to *COX-2* mRNA expression and *PIK3CA* mutations in the global TCGA cohort. **D.** Kaplan-Meier estimates of disease-free survival according to *COX-2* mRNA expression and *PIK3CA* mutations in HR+ patients. “Other” refers to low *COX-2 PIK3CA* mutated tumors and high *COX-2 PIK3CA* wild-type and mutated tumors.

We then assessed the prognostic impact of *COX-2* expression depending on the *PIK3CA* mutational status in the cohort of 446 patients. Independently of the subtype of BC and adjuvant treatment received (chemotherapy, hormone therapy, both or none) MFS was significantly better in patients with high *COX-2* expression (p=0.007, HR 1.560 [1.130-2.153]) (Figure 2A) and in patients with *PIK3CA* mutations (p=0.02, HR 1.455 [1.058-2.002]) (Figure 2B). *COX-2* expression level had no impact on MFS in the *PIK3CA* mutated patients' subgroup (Figure 2C). However in the *PIK3CA* wild-type patients' subgroup MFS was significantly better in patients with high COX-2 expression as compared to patients with low *COX-2* expression (p=0.01, HR 1.617 [1.113-2.350]) (Figure 2C). Interestingly, the same result was observed in HR^+^ tumors where *PIK3CA* mutations are clearly associated with good prognosis (p=0.0004, HR 2.377 [1.473-3.835]) [35–37]. Patients with high *COX-2* expression and *PIK3CA* wild-type had a similar MFS as *PIK3CA* mutated patients (p=0.07, HR 1.717 [0.9458-3.116]) (Figure 2D). Low *COX-2* expression and *PIK3CA* wild-type status allowed to identify patients with the worse MFS in the total cohort (p=0.0004, HR: 1.761 [1.289 to 2.405]) and among HR+ tumors (p= 0.0002, HR: 2.018 [1.397 to 2.914]) (Figure 3A and 3B). Given the limited number of triple-negative and HR^-^ERBB2^+^ cases, it was not appropriate to evaluate the prognostic impact of *COX-2* expression according to the *PIK3CA* status in these two subtypes.

*COX-2* expression and *PIK3CA* mutational status did not impact overall survival (OS) in this cohort with very long follow-up (Supplementary Figure S2).

In the TCGA validation set, high COX-2 expression was associated with a better DFS (p=0.0014, HR 2.206 [1.356-3.587]) and *PIK3CA* mutations did not have prognostic impact on DFS (Supplementary Figures S3A and S3B). After combination of these two parameters, *COX-2* expression level did not have prognostic impact in *PIK3CA* wild-type patients but high *COX-2* level expression was associated with a better DFS among mutated patients (p=0.0007, HR 4.667 [1.917-11.36]) (Supplementary Figure 3C). In the luminal subtype, high COX-2 expression was associated with a better DFS (p=0.012, HR 2.682 [1.243-5.785]) and *PIK3CA* mutations did not have prognostic impact on DFS (data not shown). Among *PIK3CA* wild-type patients, high COX-2 patients had a better DFS than low *COX-2* patients (p=0.012, HR 3.206 [1.287-7.984]) (Supplementary Figure S3D).

Similarly to Institut Curie BC cohort, low *COX-2* expression and *PIK3CA* wild-type status allowed to identify TCGA patients with the worse DFS in the entire cohort (trend, p=0.105, HR: 1.584 (0.9090 to 2.758) and among HR+ tumors (p=0.024, HR: 2.698 [1.142 to 6.376]) (Figure 3C and 3D).

In the TCGA BC cohort overall survival data showed a better prognosis for high *COX-2* patients (p=0.0011, HR 2.452 [1.428-4.208] but not for PIK3CA mutated patients (Supplementary Figure S4A and S4B). Among wild-type *PIK3CA* patients, high COX-2 patients had a better overall survival than low *COX-2* patients (p=0.018, HR 2.183 [1.146-4.157]). The same significant difference was observed in *PIK3CA* mutated patients (p=0.015, HR 3.494 [1.273-9.592]) (Supplementary Figure S4C). In the luminal subtype, high COX-2 expression was associated with a better OS (p=0.0051, HR 1.831 [0.999-3.357]) and *PIK3CA* mutations did not have a prognostic impact on OS (data not shown). Among mutated *PIK3CA* patients, high COX-2 patients had a better overall survival than low *COX-2* patients (p=0.023, HR 3.206 [1.177-8.734]) (Supplementary Figure S4D).

### Celecoxib antitumoral effect is only observed in breast tumor harboring a *PIK3CA* mutation

Since *COX-2* overexpression was associated with TNBC subtype, we chose triple-negative PDX to investigate celecoxib antitumoral effect. Moreover there is a need for targeted therapies in TNBC.

**Figure 4:**
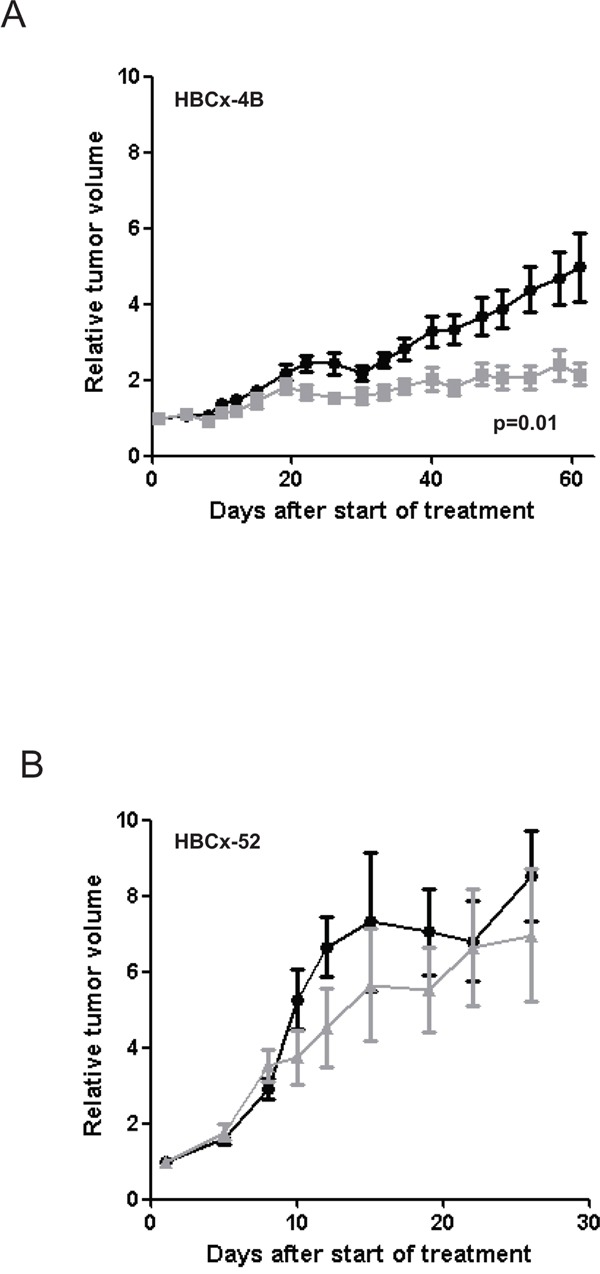
Effect of celecoxib on tumor growth of HBCx-4B and HBCx-52 PDX Tumor growth was evaluated by plotting the mean of the RTV±SD over time. **A.** HBCx-4B bearing mice were treated with celecoxib (n=18), (40 mg/kg *per os* daily five times a week). Controls (n=20) received MCT (methylcellulose 5% and 0,2% tween *per os* daily five times a week). **B.** HBCx-52 bearing mice were treated with celecoxib (n=6), (40 mg/kg *per os* daily five times a week). Controls (n=7) received MCT (*per os* daily five times a week).

In the *PIK3CA* mutated TNBC PDX model (HBCx-4B) a significant reduction in tumor volume (RTV) was observed in mice receiving celecoxib as compared to control mice from day 22 (p=0.03) and until the end of the experiment (day 61, TGI=57%, p=0.01) (Figure 4A). These results clearly showed that celecoxib induced a significant antitumor effect in tumors expressing COX-2 and harboring a *PIK3CA* mutation [31]. The TGI obtained with celecoxib in this model was near to the 60% proposed by Wang *et al*. as a cut-off for mice xenografts likely to lead to a positive clinical outcome [38]. Of note celecoxib is not a chemotherapy and was given as monotherapy in this experiment.

In contrast, in the *PIK3CA* wild-type model (HBCx-52) no significant difference in RTV was observed between the treated and control groups (day 25, p=0.94) (Figure 4B). HBCx-4B and HBCx-52 PDX models both express high *COX-2* mRNA level and COX-2 protein (Table 2). However antitumoral effect was observed in HBCx-4B model only. We can therefore ascertain that celecoxib had no effect on the tumor growth in tumors expressing COX-2 and *PIK3CA* wild-type.

### Antitumoral effect of celecoxib in *PIK3CA* mutated tumors involves phosphorylation of PI3K/AKT pathway main actors

Western-blot analysis in both PDX showed a significant decrease of COX-2 expression in the celecoxib treated group compared to controls (p=0.018 for HBCx-4B and p=0.02 for HBCx-52) confirming the pharmacological effect of this molecule. Celecoxib treatment did not affect angiogenesis as shown by the MVD assays in treated and control tumors of both PDX models (Table 5).

**Table 5:** Assessment of celecoxib treatment on angiogenesis (microvessel density) performed by ERG immunostaining

	control group	celecoxib-treated group	p value^c^
**HBCx-52**	40.4 ± 5.8	33.8 ± 9.0	p=0.35
**HBCx-4B**	23.5 ± 3.4	16.7 ± 3.0^a^	p=0.058
		18.1 ± 2.2^b^	p=0.14

Results are expressed as the mean (+/− SD) of the number of blood vessels by mm^2^

a Responders tumors

b Non-responders tumors

c T-test

**Figure 5:**
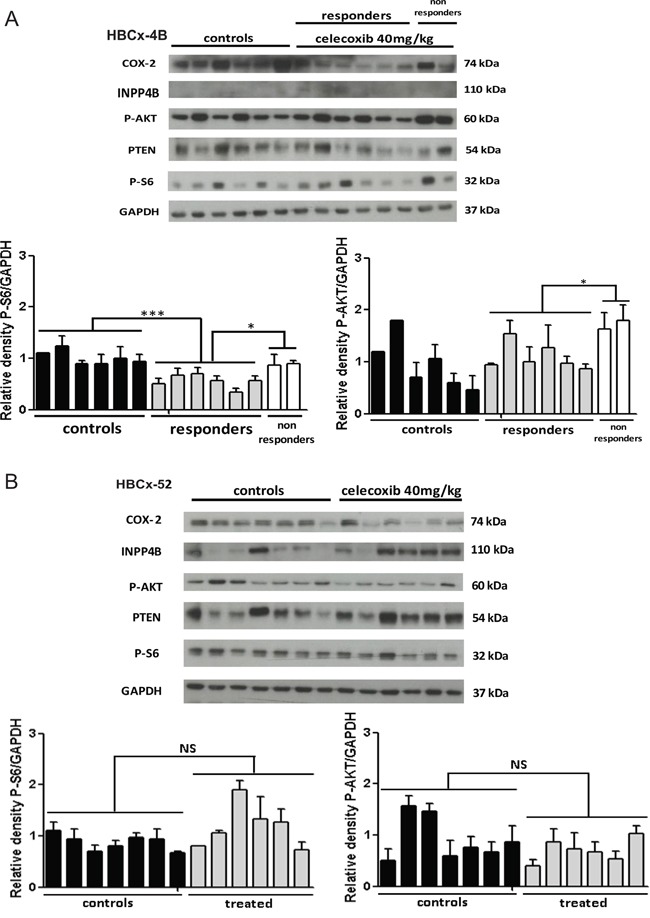
Analysis of tumors **A.** Western-blot analysis of COX-2, phospho-AKT, phospho-S6 ribosomal protein, PTEN and INPP4B in HBCx-4B control mice (n=6) and celecoxib treated mice: responders (n=6) and non-responders (n=2). **B.** Western-blot analysis of COX-2, phospho-AKT, phospho-S6 ribosomal protein, PTEN and INPP4B in HBCx-52 control mice (n=7) and celecoxib treated mice (n=6). GAPDH served as loading control. One representative blot is presented for each model. The densitometric analysis is the mean ± SEM. (n=3 experiments). For statistical analysis, treated group was compared with controls, and responders compared with non-responders. *P < 0.05 and ***P < 0.001.

In the HBCx-4B responder model, exploration of the PI3K pathway showed a significant decrease of S6 ribosomal protein phosphorylation in the treated group compared to controls (p=0.0003) (Figure 5A). We observed a significant increase in the expression of this phospho-protein in two tumors, which progressed under celecoxib as compared to responders (p=0.02) (Figure 5A). These two non-responder tumors showed also a significant increase of AKT phosphorylation by comparison to responders (p=0.02) (Figure 5A).

In HBCx-52 similar phosphorylation levels of S6 ribosomal protein and AKT were observed in controls and treated tumors (Figure 5B).

PTEN, another major component of the PI3K/AKT pathway, was not differentially expressed in these two PDX models. INPP4B expression was lost in the HBCx-4B responder model but also in several HBCx-52 tumors (Figure 5A and 5B).

## DISCUSSION

In BC, COX-2 expression level and its prognostic value have been controversial for several decades. In the present study, we observed an overexpression of COX-2 only in a small percentage of BCs, predominantly belonging to the triple-negative subtype. More importantly the under-expression of COX-2 is an independent pejorative prognostic factor. Low *COX-2* and *PIK3CA* wild-type status was identified as the worse prognostic factor for MFS in our cohort and confirmed for DFS in an independent validation set.

Several published studies reported variable expression levels of COX-2 in BCs but most of them assessed COX-2 expression at the protein level. In the present study, we applied qRT-PCR on RNA extracted from a large cohort of samples all infiltrated with more than 70% of tumor cells to assess COX-2 expression. With the exception of few cases in triple-negative subtype, we showed that the majority of BCs under-express *COX-2* mRNA or express *COX-2* mRNA at a similar level to normal breast tissue. Few studies assessed *COX-2* mRNA expression level. Contrary to our results, several authors found an overexpression of *COX-2* mRNA in BC tissues when compared to benign breast lesions [39] or breast normal tissues [2, 40]. All these results were obtained using qualitative RT-PCR in very small cohorts of BCs (13, 10 and 9 cases), which might explain the discrepancy. One report by McCarthy *et al.* using qRT-PCR, showed that the median *COX-2* mRNA expression in 45 primary invasive BC samples was not significantly different as compared to the median *COX-2* mRNA expression in 22 normal breast tissues [41]. These authors also showed that *COX-2* mRNA levels were significantly higher in estrogen (*p* < 0.02) and progesterone (*p* < 0.0001) receptor negative tumors but they did not assess the ERBB2 status of the tumors and consequently no conclusions can be drawn as for the level of mRNA expression of *COX-2* in the triple- negative subtype [41]. According to our results Kirkpatrick *et al.* using qRT-PCR found in 40 infiltrating carcinomas and 40 matched adjacent non-cancerous tissue (ANCT) that *COX-2* mRNA copy number per μg of RNA was two-fold higher in ANCT compared to the cancerous tissue (p=0.01) [42]. Using the robust and reproductive tool of qRT-PCR, our results are therefore similar to the above reports and confirm that *COX-2* mRNA overexpression is not a hallmark in all BCs.

We have demonstrated a good correlation between *COX-2* mRNA and protein level suggesting that we would expect to find the same minority overexpressed cases with an IHC-based study. This result was obtained by examining few cases using IHC and is contradictory with the majority of published studies describing COX-2 overexpression in invasive BCs [8, 11, 40, 43] but not all [44]. Like the majority of these studies we focused on COX-2 expression in epithelial cells and did not examine stroma. Recently Urban *et al.* analyzed the prognostic value of COX-2 expression not only in breast epithelial cell but also in stromal cell using different antibodies and scoring algorithms. Although they showed that COX-2 expression in stromal cells and not in epithelial cells is an independent adverse prognostic factor and is relatively insensitive to variations of antibodies used, they finally explained the variability of published results by the use of different antibodies and scoring algorithms [45]. So we prefer to remain cautious about the IHC results and it should be reminded that our conclusions concerning the prognostic value of *COX-2* expression is based on *COX-2* mRNA expression level.

Published results concerning the changes of COX-2 protein expression during the disease progression and its prognostic significance are contradictory, even in the groups working with the same type of primary antibodies [45]. Miglietta *et al.* published that COX-2 immune positivity and percentage of positive cells correlated significantly with the size, grading, extent of primary tumor and vascular invasion of carcinoma but not with biological parameters (HR and ERBB2 status). Nevertheless, for Park *et al*. there was no significant association between COX-2 over-expression and tumor size, histologic grade, and estrogen receptor expression [46, 47]. In the work of Kim *et al.* COX-2 positivity was significantly correlated with high grade, negative ER, high Ki67, luminal B and triple-negative tumors [48].

To the best of our knowledge we conducted the first *COX-2* mRNA expression prognostic impact study in a large cohort of BC patients. Under-expression of *COX-2* transcript was associated with poor prognosis and HR status but was not with other classical criteria like high grade, tumor size or lymph node status. Multivariate analysis showed that *COX-2* under-expression, high grade, higher tumor size and lymph node involvement were predictive of poor prognosis. *COX-2* under-expression should be thus considered as an independent poor prognostic factor. Moreover this under expression combined with a *PIK3CA* wild-type status allowed to identify a poor prognosis subgroup of patients who might benefit from more intensive treatment regimens. This result was confirmed with the DFS analysis in a TCGA validation cohort. High *COX-2* expression is significantly correlated with better OS of *PIK3CA* wild-type and mutated patients in the TCGA cohort but this result was not observed in our BC cohort with a longer median follow-up delay (8.6 years for the Curie cohort *versus* 28.9 months for the TCGA cohort). Interplays between COX-2 and the PI3K/AKT pathway have been already well described in colorectal cancer [49] but still need to be deciphered in BC to explain effects of their combination.

In accordance with the observations made by Liao *et al*. in colorectal cancer, our *in vivo* PDX experiments showed that celecoxib antitumoral effect was restricted to *PIK3CA* mutated breast tumors. *PIK3CA* status has never been explored in BC clinical trials assessing concomitant administration of exemestane or chemotherapy with celecoxib. Our findings led to the hypothesis that the negative results of these clinical trials might come from the fact that patients were not selected according to tumor *PIK3CA* status. Consequently a retrospective analysis of results of these trials regarding *PIK3CA* status could be very interesting. Eventually, new prospective trials combining celecoxib with hormone therapy or chemotherapy may screen patients for tumor *PIK3CA* mutations to confirm its predictive value. It is also important to underline that our *in vivo* experiments were done with TNBC PDX whereas clinical trials were designed for luminal BCs. We cannot exclude that in this subtype of BCs some other unknown factors could interact negatively with antitumoral properties of celecoxib.

There were two non-responders tumors in our *PIK3CA* mutated PDX model. The protein expression analysis on collected tumor xenografts revealed an increase of AKT phosphorylation in these two tumors. In ovarian tumors and MCF-7 breast tumor cell line COX-2 overexpression is associated with an increase of AKT phosphorylation [50, 51], which might explain resistance to celecoxib in both tumors. Antitumoral effect of celecoxib is associated with the inactivation of PI3K/AKT pathway as observed with the decrease of S6 kinase phosphorylation whereas secondary resistance is explained by AKT reactivation.

In conclusion, treatment with celecoxib may be an additional therapeutic option for patients with BCs expressing COX-2 protein and mutated for *PIK3CA whatever the level of cox-2 mRNA expression*. Thus the detection of COX-2 protein should be the only pre-requisite criteria for mutated tumors treatment with celecoxib. Noteworthy, *PIK3CA* mutation screening and COX-2 IHC staining are very easy to implement in diagnostic laboratory and could be used routinely for patient selection. These results were obtained with two PDX models only and need to be validated in a clinical trial. In this way we note that no published clinical trials with celecoxib in BC patients reported cardiac toxicity restricting the use of this FDA approved NSAID so it should not be a limiting factor for future trials.

## MATERIALS AND METHODS

### Patients

Samples of 446 primary breast tumor, excised from women treated at Institut Curie - Hôpital René Huguenin (Saint-Cloud, France) from 1978 to 2008, have been analyzed. All patients treated at Institut Curie before 2007 were informed that their tumor samples might be used for scientific purposes and had the opportunity to decline. Since 2007, patients treated at Institut Curie have given their approval by signing an informed consent. This study was approved by the local ethics committee (Breast Group of René Huguenin Hospital). The samples were immediately stored in liquid nitrogen until RNA extraction. A tumor sample was considered suitable for this study if the proportion of tumor cells exceeded 70%.

All patients (mean age 61.8 years, range 31 – 91 years) met the following criteria: primary unilateral non metastatic breast carcinoma for which complete clinico-pathological data and follow-up were available; no radiotherapy or chemotherapy before surgery; and full follow-up at Institut Curie - Hôpital René Huguenin. Adjuvant therapy was administered to 361 patients, consisting of chemotherapy alone in 87, hormone therapy alone in 175, and both treatments in 99 patients.

Estrogen receptor (ER), progesterone receptor (PR), and human epidermal growth factor receptor 2 (ERBB2) statuses were determined at the protein level by using biochemical methods (Dextran-coated charcoal method, enzyme immunoassay or immunohistochemistry) and confirmed by real-time quantitative RT-PCR assays [22, 23]. The population was divided into 4 groups according to HR (ER and PR) and ERBB2 status as follows: two luminal subtypes [HR^+^ (ERα^+^ or PR^+^)/ERBB2^+^ (n=51)], and [HR^+^ (ERα^+^ or PR^+^)/ERBB2^-^ (n=285)]; an ERBB2^+^ subtype [HR^-^ (ERα^-^ and PR^-^)/ERBB2^+^ (n=42)]; and a triple-negative subtype [HR^-^ (ERα^-^ and PR^-^)/ERBB2^-^ (n=68)]. Standard prognostic factors are shown in Table 3. Within a median follow-up of 8.6 years (range 6 months to 29 years), 164 patients developed distant metastasis. Ten specimens of adjacent normal breast tissue from BC patients (n=2) and normal breast tissue from women undergoing cosmetic breast surgery (n=8) were used as sources of normal RNA [24].

Public data of 817 breast invasive carcinomas from TCGA were used as a validation set [25]. This cohort was obtained by using www.cbioportal.org. [26, 27]. The population was divided into 5 molecular subtypes: basal (n=136), ERBB2+ (n=65), luminal A (n=415), luminal B (n=176) and normal (n=25). Median follow-up was 25 months (range 0 – 281.1 months) and 28.9 months (range 0 - 282.7 months) for disease-free survival and overall survival respectively. *COX-2* mRNA expression is expressed in z-Scores (RNA Seq V2 RSEM (RNA-Seq by Expectation-Maximization)).

### RNA extraction

Total RNA was extracted from breast tumor samples and PDX tumors by using acid-phenol guanidium as previously described [28]. RNA quality was determined by electrophoresis through agarose gels, staining with ethidium bromide, and visualization of the 18S and 28S RNA bands under ultraviolet light.

### Real-time RT-PCR

Quantitative values were obtained from the cycle number (Ct value) at which the increase in the fluorescence signal associated with exponential growth of PCR products started to be detected by the laser detector of the ABI Prism 7900 Sequence Detection System (Perkin-Elmer Applied Biosystems, Foster City, CA), using PE Biosystems analysis software according to the manufacturer's manuals.

The *TBP* gene (Genbank accession NM_003194) encoding the TATA box-binding protein (a component of the DNA-binding protein complex TFIID) was quantified as an endogenous RNA control, and each sample was normalized on the basis of its *TBP* content [22].

Results, expressed as N-fold differences in target gene expression relative to the TBP gene and termed “Ntarget”, were determined as Ntarget = 2^ΔCtsample^, where the ΔCt value of the sample was determined by subtracting the average Ct value of the target gene from the average Ct value of the *TBP* gene.

The Ntarget values of the samples were subsequently normalized such that the median of the Ntarget values for the ten normal breast tissues was 1. In tumor samples values of 3 or more were therefore considered to represent overexpression, and values of 0.3 or less were considered to represent underexpression of the 10 quantifiable mRNAs, as in previous studies [22, 29]

Primers' sequences are available on request. Agarose gel electrophoresis was used to verify the specificity of PCR amplicons. The conditions of cDNA synthesis and PCR were previously described [22].

### Western blot analysis

Proteins were extracted from frozen tumors using RIPA buffer (50 mM Tris–HCl (pH 8), 150 mM NaCl, 0.5% deoxycholic acid, 0.5% triton) supplemented with protease and phosphatase inhibitors. Proteins were separated by SDS-PAGE and then electrophoretically transferred into nitrocellulose membrane and probed using the following primary antibodies: anti-GAPDH (V18 clone, 1/20000) purchased from Santa Cruz Biotechnology, anti-COX-2 (12282, 1/1000), anti-phospho Serin 473-AKT (4060, 1/2000), anti-PTEN (9552, 1/2000), anti-INPP4B (14543, 1/2000) and anti-phospho-S6 ribosomal protein (2211, 1/8000) purchased from Cell Signaling Technology (Ozyme). Proteins were detected according to the ECL Western Blotting Analysis System procedure (GE Healthcare, Buckinghamshire, UK). The intensity of the protein bands was quantified using ImageJ software.

### Immunohistochemical staining

Patient and xenografted tumors were fixed in 10% neutral buffered formalin, paraffin embedded, and hematoxylin-eosin-saffron (HES) stained. The anti-COX-2 antibody (Dako reference M3617) and its isotypic control (Sigma reference F5636) were used on 26 primary breast tumors and 14 xenografted tumors. Staining (intensity and fraction of positive cells) was taken into consideration in the cytoplasm of epithelial cells only.

### Assays for microvessel density (MVD) on tumor tissues

Microvessels in tumor tissues were immunostained using anti-ERG antibody (reference AC-0105, clone EP111, Abcam) on 14 xenografts (HBCx-4B, control tumors n=3, celecoxib treated tumors, n=5 (3 responders and 2 non-responders), HBCx-52, control tumors n=3, celecoxib treated tumors n=3). MVD was assessed according to a method adapted of Weidner *et al*., 1991 [30]. The entire tumor section was first observed at low-power magnification (40x) to select the most vascularized areas (hotspots). Individual microvessels, immunoreactive for ERG, were counted at high–power magnification (400x) within 10 consecutive fields. In each tumor tissue, the microvessel count was expressed by mm^2^.

### *In vivo* experiments

*In vivo* studies were performed on female Swiss nude mice purchased from Charles River. Mice care and housing were conformed to the institutional guidelines as put forth by the French Ethical Committee. Human TNBC xenografted models were established as previously detailed [31, 32]. The effect of celecoxib (purchased from Pfizer) was evaluated in two PDX: HBCx-4B which presents a *PIK3CA* mutation and HBCx-52, wild-type for this gene, both expressing COX-2. A toxicity study was first performed on mice-bearing human BC xenografts which received 20 or 40 mg/kg of celecoxib by gavage five times a week. As no toxicity was observed, the dose of 40 mg/kg was retained for the next experiments. For HBCx-52, a control group (n=7) received gavage with MCT (methylcellulose 5% + 0.2% tween) five times a week and the treated group received five times a week 40 mg/kg of celecoxib (n=6). For HBCx-4B, the same groups were established: a MCT control group (n=20) and a celecoxib treated group (n=20). Tumor growth was evaluated with a calliper twice a week. Tumor growth inhibition (TGI) of treated tumors *versus* controls was calculated as the ratio of the mean relative tumor volume (RTV) in the treated group to the mean RTV in the control group at the same time. Statistical significance of TGI was calculated using the paired Student t test comparing the individual RTVs in the treated and control groups.

### Statistical analysis

Statistical analyses were performed using GraphPad Prism 5 software. The data are expressed as the mean ± SEM. The results were considered statistically significant at a p-value <0.05 (*), <0.01 (**), or <0.001 (***).

Relationships between mRNA levels and clinical parameters were identified by using non parametric tests, namely the Chi-square test, Fischer's test and the Mann-Whitney U test.

Metastasis-free survival (MFS) was determined as the interval between initial diagnosis and detection of the first metastasis. Overall survival (OS) was determined as the interval between initial diagnosis and death of any cause. Survival distributions were estimated by the Kaplan-Meier method, and the significance of differences between survival rates was ascertained with the log-rank test. The optimal cut-off value for *COX-2* mRNA expression prognostic value was determined with the AUC-ROC analysis defining “high” *COX-2* mRNA expression >0.22 and “low” *COX-2* mRNA expression <0.22. The Cox proportional hazards regression model was used to assess prognostic significance in the multivariate analysis and the results are presented as hazard ratios and 95% confidence intervals (CIs).

## SUPPLEMENTARY MATERIALS FIGURES AND TABLE


