# Supplementary material for: Combination of COX-2 expression and PIK3CA mutation as prognostic and predictive markers for celecoxib treatment in breast cancer
Source: Oncotarget. 2016 Nov 8;7(51):85124–41. doi: 10.18632/oncotarget.13200 (PMC5356723; doi:10.18632/oncotarget.13200)
Supplement: Supplementary file 1 [file oncotarget-07-85124-s001.pdf]

Combination of COX-2 expression and *PIK3CA* mutation as prognostic and predictive markers for celecoxib treatment in breast cancer

SUPPLEMENTARY FIGURES AND TABLE

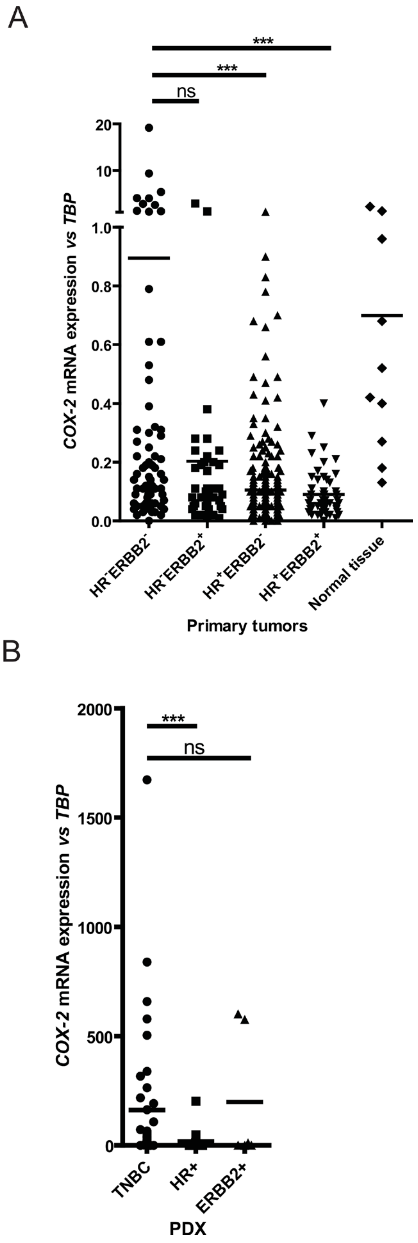

Supplementary Figure S1: *COX-2* mRNA expression in patient breast tumors and PDX models. A. *COX-2* mRNA expression levels in 446 breast tumor samples (68 HR-ERBB2-, 42 HR-ERBB2+, 285 HR+ERBB2- and 51 HR+ERBB2+) and in 10 normal breast tissues using qRT-PCR. HR for hormone receptors. *COX-2* mRNA expression in breast tumor samples and in normal tissue is expressed compared to expression of *TBP* gene. **B.** *COX-2* mRNA expression levels in 61 tumors collected on PDX (15 HR^+, 6 $ERBB2^+$ and 40 triple negative tumors). *COX-2* mRNA expression is expressed compared to expression of *TBP* gene.

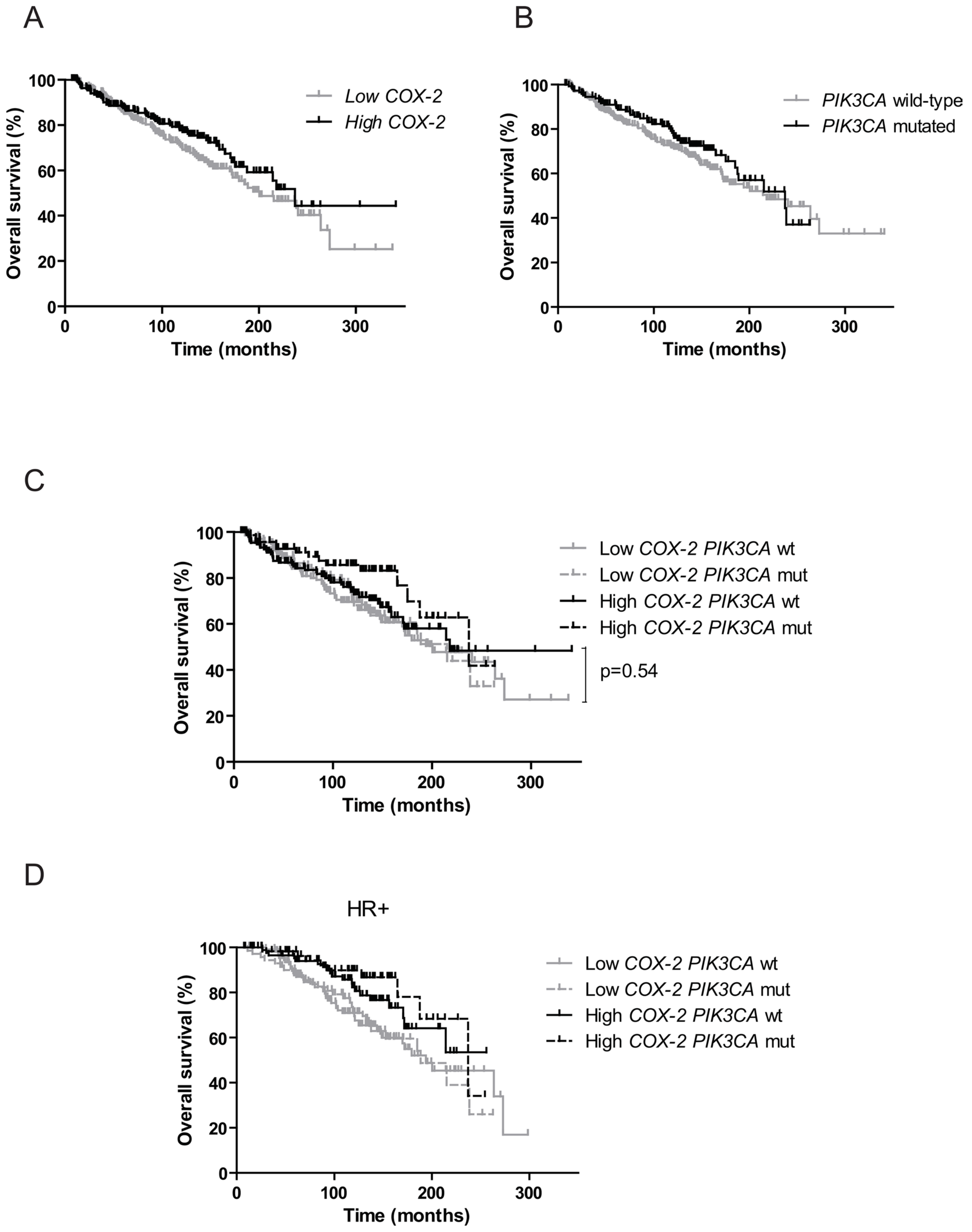

Supplementary Figure S2: Prognostic value of *COX-2* mRNA expression and *PIK3CA* mutations on patients' overall survival. A. Kaplan-Meier estimates of overall survival according to *COX-2* mRNA expression. **B.** Kaplan-Meier estimates of overall survival according to *PIK3CA* mutations. **C.** Kaplan-Meier estimates of overall survival according to *COX-2* mRNA expression and *PIK3CA* mutations in the global cohort. wt for wild-type, mut for mutated. **D.** Kaplan-Meier estimates of overall survival according to *COX-2* mRNA expression and *PIK3CA* mutations in HR+ patients.

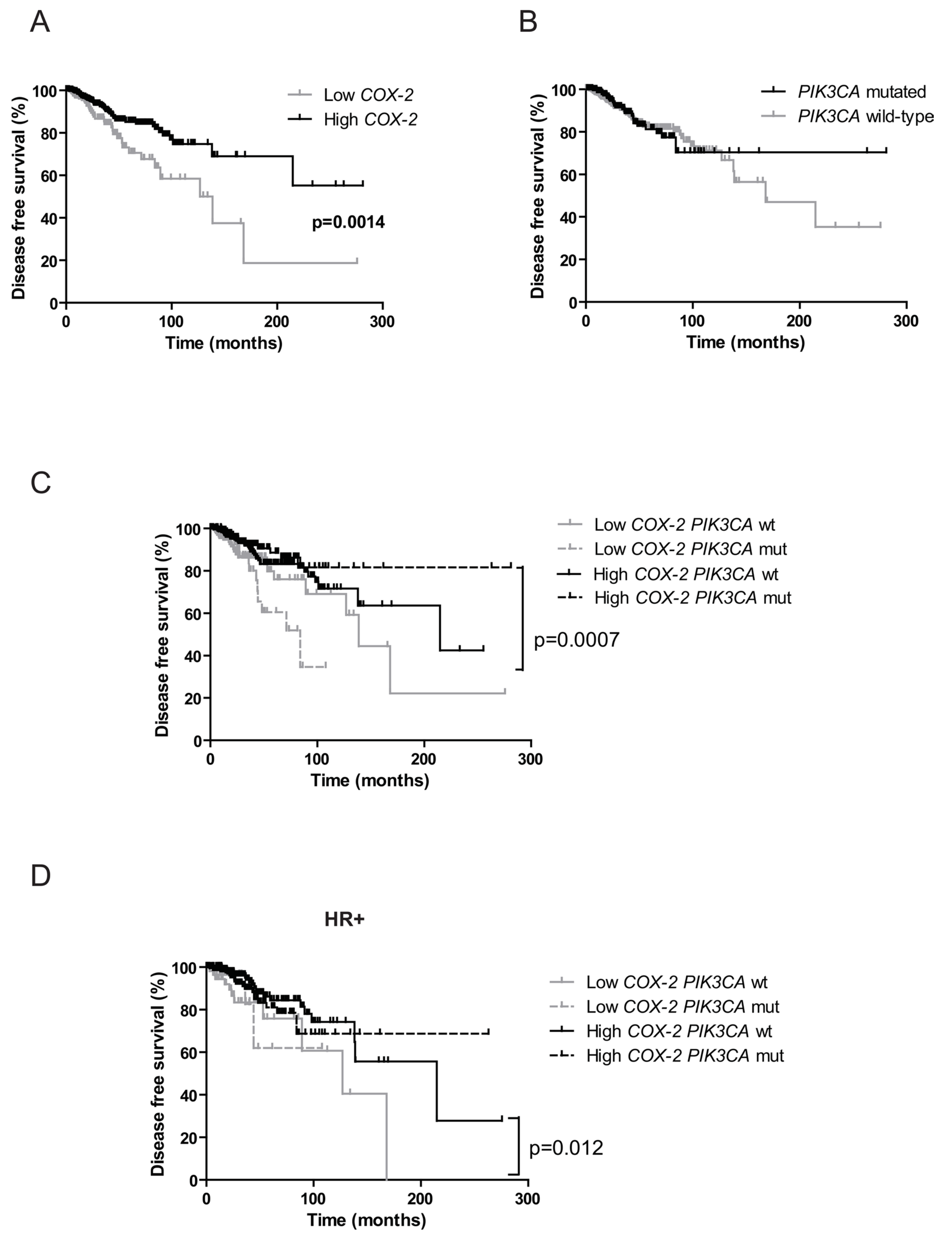

Supplementary Figure S3: Prognostic value of *COX-2* mRNA expression and *PIK3CA* mutations on patients' disease-free survival (TCGA public available data). A. Kaplan-Meier estimates of disease-free survival according to *COX-2* mRNA expression. **B.** Kaplan-Meier estimates of disease-free survival according to *PIK3CA* mutations. **C.** Kaplan-Meier estimates of disease-free survival according to *COX-2* mRNA expression and *PIK3CA* mutations in the global cohort. wt for wild-type, mut for mutated. **D.** Kaplan-Meier estimates of disease-free survival according to *COX-2* mRNA expression and *PIK3CA* mutations in HR+ patients.

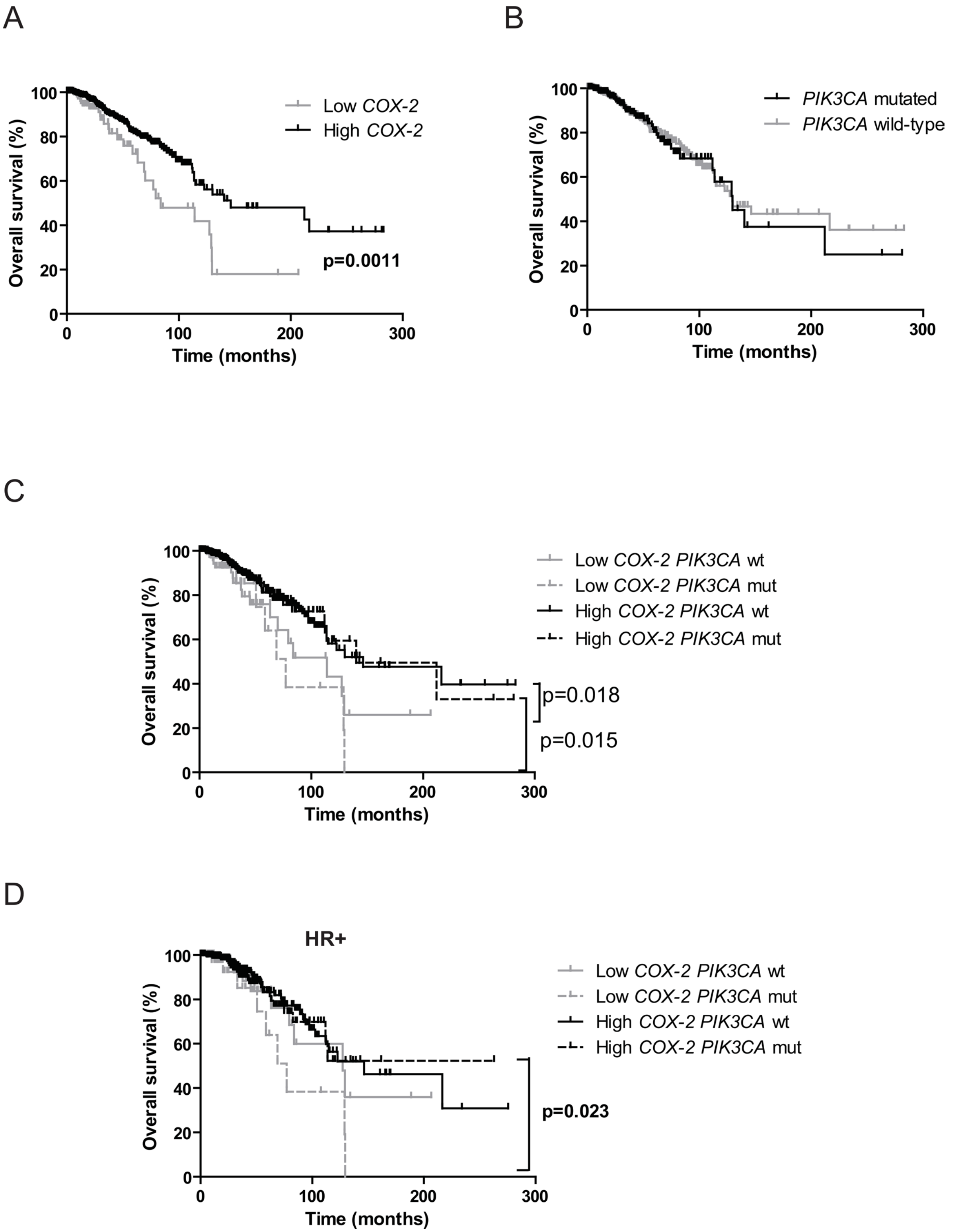

Supplementary Figure S4: Prognostic value of *COX-2* mRNA expression and *PIK3CA* mutations on patients' overall survival (TCGA public available data). A. Kaplan-Meier estimates of overall survival according to *COX-2* mRNA expression. **B.** Kaplan-Meier estimates of overall survival according to *PIK3CA* mutations. **C.** Kaplan-Meier estimates of overall survival according to *COX-2* mRNA expression and *PIK3CA* mutations in the global cohort. wt for wild-type, mut for mutated. **D.** Kaplan-Meier estimates of overall survival according to *COX-2* mRNA expression and *PIK3CA* mutations in HR+ patients.

Supplementary Table S1: Results of Cox multivariate analysis

	HR [CI 95%]	p value
**SBR histological grade**		
**I**	1	
**II**	3.16 [0.97-10.24]	0.05
**III**	3.53 [1.05-11.83]	**0.04**
**Lymph node status**		
**0**	1	
**1-3**	1.19 [0.72-1.99]	0.49
**>3**	1.90 [1.09-3.32]	**0.02**
**Macroscopic tumor size**		
**≤25**	1	
**>25**	1.63 [1.07-2.49]	**0.02**
**ERα**		
**Negative**	1	
**Positive**	0.65 [0.39-1.09]	0.11
**PR**		
**Negative**	1	
**Positive**	0.61 [0.37-1]	0.05
***PIK3CA* status**		
**wild type**	1	
**mutated**	0.8 [0.51-1.26]	0.34
***COX-2* expression**		
**Low**	1	
**High**	0.57 [0.36-0.90]	**0.01**
